# Polar Lipids from Olives and Olive Oil: A Review on Their Identification, Significance and Potential Biotechnological Applications

**DOI:** 10.3390/foods7070109

**Published:** 2018-07-10

**Authors:** Eliana Alves, M. Rosário M. Domingues, Pedro Domingues

**Affiliations:** Mass Spectrometry Centre, Department of Chemistry & QOPNA, University of Aveiro, Campus Universitário de Santiago, 3810-193 Aveiro, Portugal; mrd@ua.pt (M.R.M.D.); p.domingues@ua.pt (P.D.)

**Keywords:** authentication, bioactive, by-product, glycolipid, lipidomics, mass spectrometry, phospholipid, traceability

## Abstract

Polar lipids are minor components of olives and olive oil and include a myriad of molecules such as phospholipids and glycolipids. Even though sensitive and high-resolution analytical approaches have been used to unveil the polar lipidome of these matrices, new insights on their composition are needed. In this review, we will describe the findings on the identification and characterization of polar lipids from olives and olive oil and the underlying analytical challenges. The significance of polar lipids will also be discussed as potential markers of identity and traceability of olives and olive oil and in detecting adulteration of olive oil. Their potential impact on nutrition and health will be presented as a valuable source of bioactive compounds and as promising ingredients for different uses from olive-derived industrial by-products.

## 1. Introduction

For millennia, olive oil has been an essential ingredient in the Mediterranean diet, as a food source of healthy fat. It is produced mostly by Spain, Italy, Greece and by other countries of Southern Europe and North Africa [1]. Nowadays, olive oil’s economy has gained global importance, especially in gourmet cuisine, and its production has been extended to North and South Americas, Australia and Asia [1].

The increasing investment in the development of olive groves in these regions has been boosted by the benefits of olive oil’s consumption which is directly related to its composition. Olive oil is mainly composed of triacylglycerols (Ca. 98%) [2], primarily consisting of monounsaturated fatty acids, acknowledged for improving several cardiovascular risk factors [3]. In addition to the primary compounds, high-quality olive oils, such as virgin olive oils (VOOs), possess a plethora of minor components in the remaining 2% of their composition [2]. Some of the minor components confer distinct features to olive oil in terms of sensorial attributes and health benefits [4,5], and some components can be used for providing a chemical identity to olive oil [6]. 

Polar lipids are a group of minor components of olive oil [2]. The isolation, identification, and characterization of the minor components, such as polar lipids, might be essential to provide a molecular fingerprint for traceability and authenticity purposes [7]. The profiling of the major chemical components, such as triacylglycerols and total fatty acids, is insufficient to discriminate olives or olive oils, per se, and the simultaneous analysis of minor components is necessary [8]. VOOs are very susceptible to fraud and to tampering with other oils, as lower grade olive oils [9,10]. With recent analytical developments, new fast and sensitive methods have been claimed to evaluate olive oil’s authenticity [11]. Therefore, it has become urgent to find foolproof analytical approaches and molecular markers to reveal a specific chemical identity for olives and olive oil and to detect adulterated olive oil [10]. Polar lipids have been suggested as promising molecular markers of identity [12,13]. Some research has been carried out towards their identification in olives and olive oil, mainly through mass spectrometry (MS)-based approaches, but there is still much to be done. 

Another topic concerning olives’ and olive oil’s polar lipids is their positive impact on human nutrition and health, which has been little exploited [14,15]. Additionally, in recent years, polar lipids from olive-derived industrial by-products, such as olive seeds and olive pomace, have been studied as alternative sources of bioactive lipids. The new applications of polar lipids would favor the sustainable use of olive’s industrial by-products and make them attractive from the biotechnological standpoint.

## 2. Identification of Polar Lipids from Olives, Olive Oil, and Their Industrial By-Products

**Figure 1:**
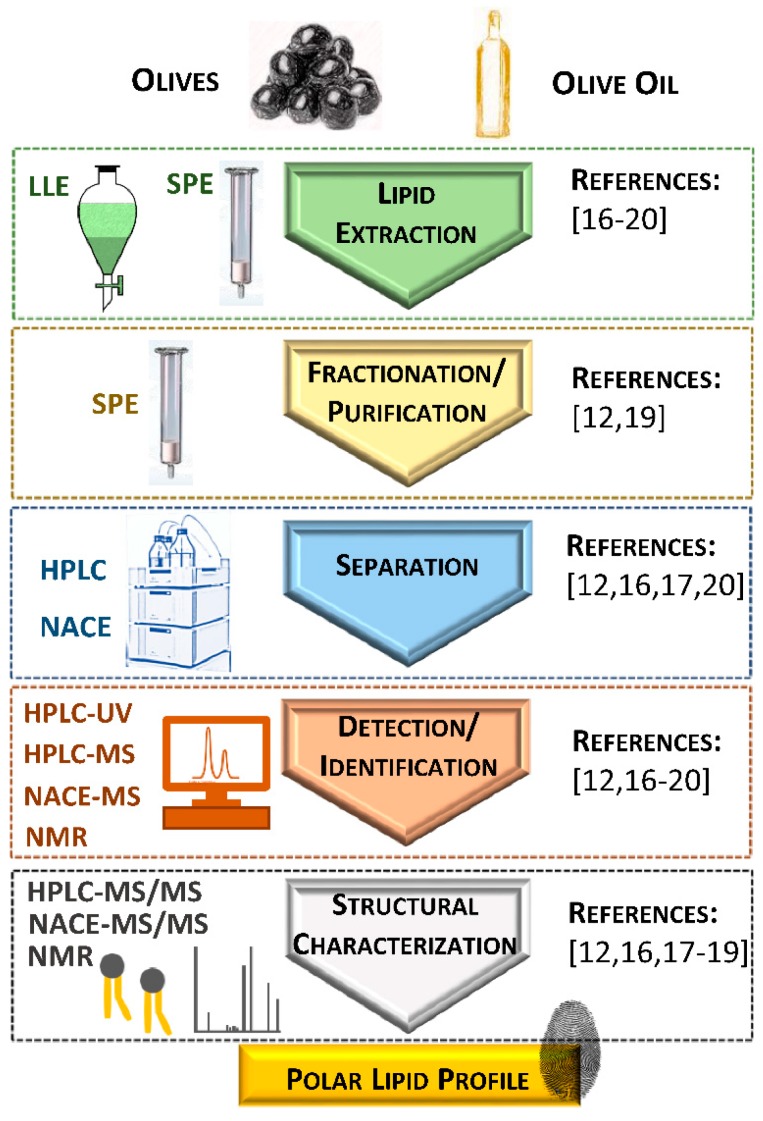
Schematic representation of the methodological approaches used for studying polar lipids from olives and olive oil. Abbreviations: HPLC, high-performance liquid chromatography; HPLC-MS, high-performance liquid chromatography coupled to mass spectrometry; HPLC-MS/MS, high-performance liquid chromatography coupled to tandem mass spectrometry; HPLC-UV, high-performance liquid chromatography with ultraviolet detector; LLE, liquid/liquid extraction; NACE, non-aqueous capillary electrophoresis; NACE-MS, non-aqueous capillary electrophoresis coupled to mass spectrometry; NACE-MS/MS, non-aqueous capillary electrophoresis coupled to tandem mass spectrometry; NMR, nuclear magnetic resonance; SPE, solid-phase extraction.

The identification of polar lipids in olives and olive oil is a difficult task since they are minor components and include a broad range of lipid classes. Different analytical approaches have been used to unravel the polar lipidome of these matrices. The lipidomic workflows included lipid extraction, fractionation, analysis and quantification (Figure 1).

Liquid/liquid extraction (LLE) has been used for extracting polar lipids from olives and olive oil. The most commonly used LLE methods were a modified Bligh and Dyer method [16], a modified Folch method [17] and a sequential LLE method developed by Galanos and Kapoulas [17,18,19]. Solid-phase extraction (SPE), using aminopropyl-bonded silica as sorbent, was recently used to obtain polar lipid-enriched fractions directly from olive oil [12]. There are other emerging extraction techniques that can be used for oil extraction from olives, such as ultrasound or microwave or CO_2_-assisted techniques, but these approaches have not yet been reported for the analysis of polar lipids in olives or olive oil.

After extraction, the total lipid extract can be fractionated to obtain polar lipid-enriched fractions or specific polar lipid classes. Polar lipid-enriched fractions were obtained using SPE cartridges with different stationary phases (silica and diol-bonded silica) after olive oil’s LLE [19].

^31^P nuclear magnetic resonance (NMR) spectroscopy [18] and non-aqueous capillary electrophoresis (NACE) coupled with MS [17] were used for the detection and characterization of the phospholipid classes of olive oil.

The separation of the polar lipid classes obtained from olive oil was carried out by high-performance liquid chromatography (HPLC) coupled to different detectors, as ultraviolet detectors (HPLC-UV) [20] or mass spectrometers (HPLC-MS) [12,16,19]. The structural characterization of the polar lipid molecules, namely the polar head and fatty acyl composition, has been achieved by using tandem MS (HPLC-MS/MS in [12,16,19] and NACE-MS/MS in [17]).

**Figure 2:**
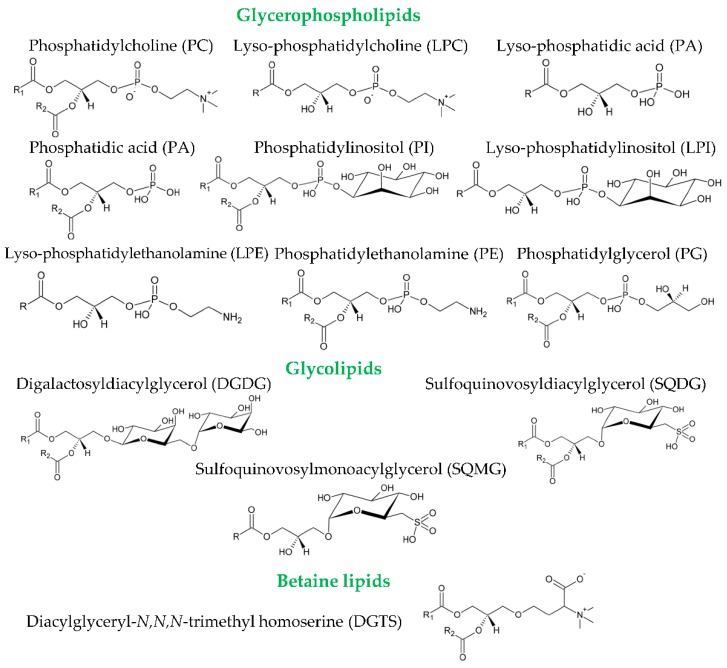
Chemical structures of the classes of glycerophospholipids and glycolipids identified in olives and olive oil. Polar lipids include a broad range of molecules. Phospholipids are divided into two main classes depending on whether they contain glycerol (glycerophospholipids) or a sphingosyl (sphingophospholipids) backbone. Glycerophospholipids, besides the glycerol backbone, contain a polar phosphorus moiety. They derive mainly from *sn*-1,2-diacylglycerols and, thus, contain structures that are based on 3-*sn*-phosphatidic acid [31]. These lipids are grouped into classes based on the composition of their polar head group that is attached to the phosphate residue in *sn*-3 position. The polar head may be an amino acid, an amino-alcohol, a carbohydrate or another functional moiety. Each head group class is further differentiated into subclasses based on the *sn*-1 and *sn*-2 substituents on the glycerol backbone [31]. Glycolipids also include a wide variety of structures. These structures consist in acylglycerols (in the case of glycosylglycerides and sulfolipids) joined to a carbohydrate moiety by a glycosidic linkage at the *sn*-3 position [31]. Betaine lipids are ether-linked glycerolipids containing a betaine moiety. These lipids contain a polar group linked by an ether bond at the *sn*-3 position of the glycerol moiety, with the fatty acids esterified in the *sn*-1 and *sn*-2 positions [31]. 1,2-diacylglyceryl-3-O-4′-(*N*,*N*,*N*-trimethyl)-homoserine (DGTS) have been commonly found in lower plants, algae, fungi, and bacteria [32]. R, R1, and R2 represent fatty acyl chains.

The analytical approaches used so far (Table 1) showed different results. In olive fruits, the polar lipidome has been studied in the oil extracted both from the pulp and the seed. Bianco et al. (1998) identified glycolipids in the olive pulp, namely digalactosyldiacylglycerols as DGDG(18:3/18:3) and DGDG(18:1/18:3) [20]. Montealegre et al. (2013) analyzed the glycerophospholipid profile of olive fruits from different Spanish cultivars and regions [17]. The glycerophospholipids identified in the olive pulp and in the seed included phosphatidic acid (PA), lyso-PA, phosphatidylethanolamine (PE), lyso-PE, phosphatidylcholine (PC), phosphatidylinositol (PI) and phosphatidylglycerol (PG) [17] (Figure 2).

Olive oil has been shown to possess several classes of glycerophospholipids (Figure 2), but the presence and amount of each class vary considerably among studies (Table 1). Authors found that the main glycerophospholipid class is PG [16], PE [17], PA [18], or lyso-PA [19]. In Greek VOOs, glycerophospholipids were identified and quantified by ^31^P-NMR [18]. PA, PI, and lyso-PA were the major classes identified, but PC, PE, and lyso-PI were also found. PG was only detected in one olive pomace oil that showed higher diversity and concentration in the glycerophospholipid classes: PA, lyso-PA, PI and lyso-PI [18]. The MS-based approaches used by other researchers also led to different results. The glycerophospholipid profile of a commercial Tunisian olive oil was composed by PG, PA, PI, PE, and PC (by descending order of abundance) [16]. In a monovarietal commercial olive oil from the Spanish cultivar Arbequina, the glycerophospholipid classes identified were, by descending order, PE, PG, PC, lyso-PE and lyso-PA [17]. The glycerophospholipid classes from an Italian VOO blend with Leccino, Frantoio, and Picholine cultivars were PG, PA, lyso-PA, PI, PC and lyso-PC [19]. Calvano et al. (2012) identified different molecular species of PC in one commercial olive oil [13]. Molecular species of PA, PE, PG, PC, and PI were identified in Portuguese commercial olive oils, as well as other lipid molecules not identified previously in this matrix [12]. Glycolipids as DGDG(18:3/18:3) and DGDG(18:3/18:1) were identified in one Italian olive oil [20]. Table 1 summarizes the main results of each of these works. All the molecular species of glycerophospholipids and glycolipids identified by the MS-based lipidomic approaches, both in olives and olive oil, are listed in Table 2 and Table 3, respectively.

The concentration of glycerophospholipids in olive oils has been estimated by measuring the total phosphorus amount [21] using reference methods. For absolute quantification authors used ^31^P-NMR spectroscopy [18] and HPLC-MS [19]. Glycerophospholipids in olive oil are in parts per million (mg kg^−1^ of olive oil): 21 to 124 [21]; 11 to 157 [18] and 3.29 to 8.25 [19].

### Analytical Challenges in Identifying Polar Lipids in Olives and Olive Oil

The results provided by the identification and characterization of the mentioned studies are motivating but not comparable. A systematic analysis of polar lipids in these matrices has not been performed yet, and there are no official methods for their characterization.

There are several advantages of using HPLC-MS approaches. They allow a fast and reproducible analysis, in a short time-frame [16,17], with high sensitivity [19]. At the same time, it provides detailed structural information about the polar head and fatty acyl composition of each glycerophospholipid class, by the analysis and interpretation of the MS/MS data [16]. However, some analytical bottlenecks of this methodology include: chemical noise in the MS/MS spectra and unachievable MS/MS identification of the fatty acyl chains for all glycerophospholipids, due to their low amount in the samples [16]; absence of fragmentation data, possibly due to their low concentration and the complexity of the matrix [17]; and the need for further validation of quantification values with absolute quantitative methods [17].

In addition to MS-based approaches, NMR spectroscopy is a potentially valuable technique for analyzing phospholipids in olive oil [22]. Compared to HPLC-MS, NMR and specifically ^31^P-NMR, has higher selectivity, ease of performance and faster analysis. Another advantage of using ^31^P-NMR is that glycerophospholipids give a single signal in the spectrum, while different molecules of these lipids are characterized by specific resonance frequencies derived from their distinct chemical structures. As such, there is no need to separate the components in the sample before analysis [18], as in HPLC-MS. Even so, ^31^P-NMR high-resolution spectra were hampered by the formation of aggregates and electrostatic complexes with ions in solution [18]. The fatty acyl composition of the phospholipids could only be estimated since proton signals are common to the various fatty acyl chains attached to *sn*-1 and *sn*-2 positions of glycerol in the phospholipid molecules [23]. Long spectra acquisitions (one hour) were needed to achieve a reasonable signal to noise ratio, due to the low concentration of glycerophospholipids in olive oil [18]. The authors needed 100 g of olive oil to carry out the experiments and to obtain the glycerophospholipid profile, while others, using LC-MS/MS approaches, demanded 1 g [12] to 50 g [17]. Besides, the ^31^P-NMR approach cannot identify glycolipids in olive oil and these lipids also make up the polar lipid pool of this matrix [12,20].

## 3. The Importance of Studying Polar Lipids from Olives and Olive Oil

### 3.1. Authentication, Traceability, and Detection of Adulteration

Olive oil’s chemical composition depends on both geographical and botanical origin of olives. It also varies with pedo-climatic, environmental, agricultural and technological conditions, which gives rise to a unique product with distinct features. High-quality olive oils, as VOOs, have a specific chemical fingerprint, but it has been difficult to assign an identity that can differentiate VOOs from other olive oils. Consequently, the authenticity of VOOs can be at risk during the olive oil chain production, and ultimately, it may lead to fraud or adulteration. To date, it has not been possible to provide an identity to each olive oil. 

Some research groups studied olive oil’s polar lipids to address identity, traceability, and authenticity issues. A first approach based on the relative abundances of the glycerophospholipids allowed to distinguish the botanical and geographical origin of olive fruits. The olives samples used for the study were from different Spanish varieties (Empeltre, Lechín de Sevilla, and Arbequina) from the same region (Córdoba), and from the same variety (Arbequina) from different regions (Toledo, Córdoba and Jaén) [17]. There were variations on the relative abundance of the polar lipid classes identified in the seeds and in the pulp, and among different olive pulps from different cultivars. Some classes that were found in the pulp (lyso-PE and PE) were not detected in the respective seed and not all the classes were detected in all olive pulp samples. For instance, lyso-PA was not detected in Empeltre variety from Córdoba [17]. In another work, it was found that each olive oil seems to reveal a unique polar lipid profile [12], showing that each olive oil had a different PC profile and one olive oil had specific polar lipid molecular species, not detected in the other samples. These findings are a promising start for future research on olive oil’s identity and traceability. 

In the case of adulteration, it is possible to detect adulterated olive oil with levels as low as 1 to 5% of hazelnut oil, based on glycerophospholipids as biochemical markers [13]. This is a significant advantage since adulteration of extra VOO with hazelnut oil cannot be straightforwardly detected by well-established techniques because these oils have similar triacylglycerol, fatty acid, and sterol profiles [24].

Even though there are still methodological bottlenecks in studying polar lipids from olives and olive oil, these lipids are considered as new important biochemical markers. A phospholipid profile has been suggested to be included in a flowchart to detect the presence of a specific adulterant in olive oils [10].

### 3.2. Nutrition and Health

It has been widely acknowledged that olive oil’s intake, either within the Mediterranean diet or alone, has a positive impact on human health [25,26]. Nevertheless, little is known about the bioactivity or health benefits of phospholipids and glycolipids from olives and olive oil. A few in vivo and in vitro studies revealed anti-cancer or cancer-preventative effects of food glycolipids [27,28,29], as well as anti-inflammatory effects in arthritis and osteoarthritis [27,28,29,30]. Olive fruits possess glycolipids (DGDG) in a concentration of 280 mg kg^−1^ [20], but their bioactivity remains to be studied.

Some studies evaluated the bioactive properties of the polar lipid fraction of olive oil and olive pomace and revealed that they possess anti-thrombotic and anti-atherosclerotic activities by inhibiting platelet aggregation. This inhibition was assigned to inhibitors or antagonists of platelet aggregation factor (PAF) [15], that were further identified in olive oil as a glycerol glycolipid [14]. Olive pomace’s polar lipids also inhibited platelet aggregation in vitro [15]. The most potent antagonist was identified as a glycerylether-*sn*-2-acetyl glycolipid, structurally similar to the one previously identified in olive oil [15]. Olive pomace’s polar lipids revealed a higher potency than olive oil’s polar lipids in inhibiting PAF-induced aggregation, as well as specific PAF binding [15]. Thus, olive pomace’s polar lipids were suggested to be used as a dietary supplement in the prevention of the progression of atherogenesis [33].

There is some scientific evidence that the polar lipid fraction from olive oil and olive pomace possess anti-thrombotic and anti-atherosclerotic activity mediated by PAF. Nevertheless, more studies are needed to elucidate the chemical structure of the bioactive lipids to understand the mechanisms of action and to determine the concentration of these compounds in olive oil or olive-pomace to observe an in vivo effect in human beings.

## 4. Potential Biotechnological Uses of Polar Lipids from Olives’ and Olive Oil’s Industrial By-Products

Olive oil mills and pitted table olives’ producing industries generate several by-products, such as olive pomace and olive stones. These by-products can be recovered to create novel value-added products. In the case of polar lipids, their concentration is tens to hundred times higher in olive pomace oil [18] and olive seed oil [34], comparatively to olive oil [18,21]. Thus, polar lipids from olive pomace and olive seeds have been regarded as potentially useful from the nutritional and biotechnological standpoints and have been suggested for several novel industrial applications.

Olive pomace was proposed as the new promising lipid source for the sustainable production of animal feeds, namely functional fish feeds, feed for aquaculture fish and as an ingredient for inclusion in animal feedstocks [35]. Olive pomace after stoning has been extensively studied in mammal’s species as feed integration for improving the nutritional and nutraceutical properties of their meat as well as their milk and derived cheese [36,37,38,39,40]. Other studies carried out on fish species revealed that polar lipids from olive pomace oil [41] provide high nutritional value for fish feed [35] and increase fish cardio-protective properties [42]. The later studies carried out on fish fed with fish oil containing 4% of olive pomace indicated that the lipid fractions containing polar lipids had inhibitory activity against PAF-induced platelet aggregation [42]. Further research is needed on the bioactive properties of olive pomace and olive pomace oil for animal feed purposes and to identify the molecules within the polar lipid fraction responsible for such activity.

Other residues resulting from table olives’ production are the stones that contain the seeds. The economic potentialities of olive seeds and olive seed oil have been explored in the last few years, primarily by the industry [43]. 

Olive seed oil has 0.1% of phospholipids [34] and may have diverse technological uses in the soap, cosmetics and pharmaceutic industries [34]. Phospholipids from olive seeds can also be used for lecithin production in the agri-food industry [34]. Food derived phospholipids have several biomedical applications, for instance, as emulsifiers in pharmaceuticals and for the preparation of liposomes for cosmetics and drug delivery [44,45].

The potential biotechnological applications of the olive-derived by-products highlight the valuable alternatives that underlie the table olive’s and olive oil’s industries. However, more research is needed to characterize the polar lipidome, its health benefits and the cost-benefit of being extracted from these by-products.

## 5. Conclusions and Future Perspectives

**Figure 3:**
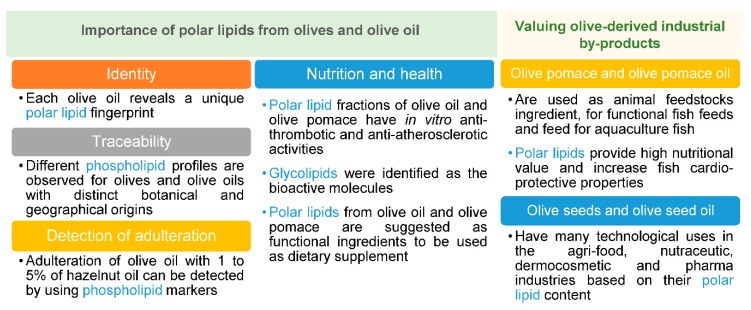
Resume on the importance of polar lipids from olives, olive oil, and their by-products.

Glycerophospholipids, glycolipids and betaine lipids were identified in olives and olive oil, but the identification of the lipidome of these foodstuffs is far from being fully covered. Distinct analytical approaches have been carried out to isolate and characterize the polar lipidome from these matrices, but those relying on NMR and MS have been the most successful. The diversity of polar lipid classes, the number of molecular species, and their ability to provide a molecular fingerprint for olives and olive oil claims for further research to achieve a standardized methodology for polar lipid identification. The study of polar lipids from olives and olive oil is essential for providing new insights into their quality, identity, authenticity, and traceability. The identification of polar lipids using the most modern technologies, as mass spectrometry coupled with liquid chromatography in a lipidomic approach, represent the most promising methodology to fulfill that goal. Simultaneously, it is also an innovative research opportunity in this field, as it can bring new inputs to the identity of these food matrices and their recognition as valuable components with health benefits. Few studies reported that polar lipids from olive oil and olive pomace possess bioactive properties, but this research field is still in its infancy. Polar lipids from olive pomace and olive seeds are being envisioned as promising ingredients to be recovered from olives’ and olive oil’s industrial processing for several biotechnological uses (Figure 3). Additionally, more information is needed on the polar lipidome of olive-derived industrial by-products, namely olive pomace, olive seeds, and their oils, to promote their recycling and reuse. Based on their polar lipids, novel value-added products and formulations can be conceived as important sources of components with biological activity. The wide range of biotechnological applications for these lipids recovered from olive’s and olive oils’ by-products include the feed, pharmaceutical, nutraceutical and dermocosmetic industries. Therefore, further investigation of the polar lipidome will foster the knowledge, valorization and sustainable use of these natural resources.

## Figures and Tables

**Table 1:** Summary of the polar lipid classes identified and quantified in olives and olive oils in different studies.

Reference	Sampling		Analysis		Polar Lipid Classes
	Type of Sample	Amount of Sample	Extraction	Method	
[20]	Olive fruit and olive oil from varieties Carolea and Ottobratica, both from Calabria region (Italy)	Olive fruit (250 g); olive oil (10 mL)	Glycosidic fraction in olive fruit: ethanol and “charcoal method”; glycosidic fraction in the aqueous phase of olive oil: ethyl acetate/dichloromethane (1:1 by volume) and water	HPLC-UV (µ-Bondapak C18 column)	DGDG
[16]	Tunisian commercial olive oil	Not said	Modified Bligh and Dyer method	HPLC-MS/MS (diol column)	PG (63%), PA (12%), PI (11%), PE (9%), PC (5%)
[18]	Greek virgin olive oil, refined olive oil and olive pomace oil from local cooperatives (7 regions and 5 cultivars)	100 g	According to Galanos and Kapoulas (1962)	^31^P-NMR	PA, lyso-PA, lyso-PI, PI, PG (PG only in pomace oil), PC and PE (these two only in virgin olive oil).
[17]	Olive pulp and olive stone from Spanish Arbequina variety from three geographical regions (Córdoba, Jaén, and Toledo) and two Spanish varieties (Empeltre and Lechín de Sevilla) from the same region (Córdoba); commercial monovarietal extra virgin olive oil from Arbequina variety	Olive pulp or stone (2.5 g); olive oil (50 g)	PL from olive pulp and stone: modified Folch method; PL from olive oil: LLE according to Galanos and Kapoulas (1962)	NACE-ESI-MS and MS/MS	Olives (stone and pulp studied independently): PA (54−82%), PE (4−16%), PC (3−9%), lyso-PE (1.3−18%), PI (4.4−8%), PG (3.7−6.3%), and lyso-PA (0.1−0.2%).
					Olive oil: PE (42%), PG (38%), PC (15%), lyso-PE (4.5%), and lyso-PA (0.2%)
[19]	Italian olive oil blend (Leccino, Frantoio and Picholine varieties) from a local mill of Emilia Romagna region (Italy)	100 g for LLE; 40 g for SPE	LLE according to Galanos and Kapoulas (1962) followed by SPE (diol and silica). PL eluted with methanol and chloroform/methanol/water (3:5:2 by volume)	HPLC–ESI-qTOF-MS (HILIC column)	Diol extracted veiled extra virgin olive oil (mg kg^−1^): lyso-PA (4.23), lyso-PC (1.21), PI (1.03), PC (0.90), PA (0.81), PG (0.07). Crystallized veiled virgin olive oil (mg kg^−1^): lyso-PA (1.15), lyso-PC (0.87), PC (0.74), PI (0.48), PA (0.14)
[12]	Portuguese commercial extra virgin and virgin olive oils	1 g	PL extracted by SPE (aminopropyl columns) and eluted with acetonitrile: ammonium hydroxide (95:5 by volume)	HPLC-ESI-ion trap-MS/MS (HILIC column)	PA, PE, PG, PC, PI, SQDG, SQMG, DGTS

Legend: DGDG, digalactosyldiacylglycerol; DGTS, diacylglyceryl-N,N,N-trimethylhomoserine; HILIC, hydrophilic interaction liquid chromatography; HILIC-ESI-MS/MS, hydrophilic interaction liquid chromatography coupled to electrospray ionization tandem mass spectrometry; HPLC, high-performance liquid chromatography; HPLC-ESI-qTOF-MS, high-performance liquid chromatography coupled to electrospray ionization-quadrupole time-of-flight mass spectrometry; HPLC-UV, high-performance liquid chromatography with ultraviolet detector; HPLC-MS/MS, high-performance liquid-chromatography coupled to tandem mass spectrometry; LLE, liquid/liquid extraction; MS/MS, tandem mass spectrometry; NACE-ESI-MS, non-aqueous capillary electrophoresis coupled to electrospray ionization mass spectrometry; NMR, nuclear magnetic resonance; PA, phosphatidic acid; PC, phosphatidylcholine; PE, phosphatidylethanolamine; PG, phosphatidylglycerol; PI, phosphatidylinositol; PL, polar lipid; SPE, solid-phase extraction; SQDG, sulfoquinovosyldiacylglycerol; SQMG, sulfoquinovosylmonoacylglycerol.

**Table 2:** List of glycerophospholipid molecular species identified in olives and olive oil through mass spectrometry-based lipidomic approaches.

Reference	Olive Fruit and/or Oil	Molecular Species (C:N)	Fatty Acyl Chains (C:N)	[M + H]^+^ *m*/*z*	[M + Na]^+^ *m*/*z*	[M + K]^+^ *m*/*z*	[M − H]^−^ *m*/*z*	[M + HCOO]^−^ *m*/*z*	[M + CH_3_COO]^−^ *m*/*z*
[19]	Oil	LPA(16:1)	16:1				407.2		
[19]	Oil	LPA(18:1)	18:1				435.3		
[19]	Oil	LPC(18:1)	18:1					566.3	
[19]	Oil	LPC(18:2)	18:2					564.3	
[19]	Oil	PA(34:1)	16:1/18:0				673.5		
[17]	Fruit	PA(36:0)	18:0/18:0				703		
[17]	Fruit	PA(36:1)	18:1/18:0				701		
[19]	Oil	PA(36:2)	18:0/18:2				699.5		
[12]	Oil	PA(38:2)	18:1/20:1 and 18:0/20:2 and 18:2/20:0				727.2		
[12]	Oil	PC(32:0)	16:0/16:0	734.5					
[12]	Oil	PC(32:1)	16:0/16:1 and 14:0/18:1	732.4	754.5				
[12]	Oil	PC(32:2)	16:1/16:1 and 14:1/18:1	730.4	752.4				
[12,19]	Oil	PC(34:1)	16:0/18:1 and 16:1/18:0 and 14:0/20:1 and 14:1/20:0	760.5	782.5	798.5		804.6	818.2
[12,19]	Oil	PC(34:2)	16:1/18:1 or 16:0/18:2 and 14:0/20:2	758.5	780.5	796.5		802.6	
[12]	Oil	PC(34:3)	16:1/18:2 and 14:0/20:3, 16:0/18:3 and 16:1/18:2	756.5	778.5	794.5			
[12,17]	Oil and fruit	PC(36:1)	18:0/18:1	788.5		826.5			
[12]	Oil	PC(36:2)	18:1/18:1 or 18:0/18:2 or 16:0/20:2 or 16:1/20:1	786.5	808.6	824.5			
[17]	Fruit	PC(38:5)	20:2/18:3	809					
[12]	Oil	PC(O-34:2)	O-16:0/18:2 and O-16:1/18:1		766.4				
[12]	Oil	PC(O-34:3)	O-16:0/18:3		764.4				
[12]	Oil	PC(O-36:1)	O-18:1/18:0 and O-16:0/20:1		796.6				
[12]	Oil	PC(O-36:3)	O-18:0/18:3 and O-18:1/18:2		792.4				
[12]	Oil	PE(34:1)	16:0/18:1 and 16:1/18:0				716.3		
[17]	Fruit	PE(38:2)	20:2/18:0	773					
[12]	Oil	PG(32:0)	16:0/16:0				721.5		
[17]	Fruit	PG(34:0)	16:0/18:0				749		
[12,17,19]	Fruit and oil	PG(34:1)	16:0/18:1		771.5		747.5		
[17]	Fruit	PG(36:1)	18:1/18:0				775		
[17]	Fruit	PG(36:2), PA(42:7)	PG(18:1/18:1)		797.5	813.5	773		
[17,19]	Fruit and oil	PI(34:0)	16:0/18:0				837.6		
[17,19]	Oil and fruit	PI(34:1)	16:0/18:1				835.6		
[12]	Oil	PI(34:1-OH)	16:0/18:1-OH				851.4		
[19]	Oil and fruit	PI(34:2)	16:1/18:1 and 16:0/18:2				833.6		
[19]	Oil and fruit	PI(34:3)	16:1/18:2				831.5		
[17]	Fruit	PI(36:1)	18:0/18:1				863		
[12,17]	Oil and fruit	PI(36:3)	18:2/18:1				859.2		

(C:N) indicates the number of carbon atoms (C) and double bonds (N) in the fatty acyl side chains. Legend: LPA, lyso-phosphatidic acid; LPC, lyso-phosphatidylcholine; PA, phosphatidic acid; PC, phosphatidylcholine; PE, phosphatidylethanolamine; PG, phosphatidylglycerol; PI, phosphatidylinositol.

**Table 3:** List of glycoglycerolipid and betaine lipid molecular species identified in olive oil through mass spectrometry-based lipidomic approaches.

Reference	Molecular Species (C:N)	Fatty Acyl Chains (C:N)	[M − H]^−^ *m*/*z*	[M + H]^+^ *m*/*z*
[12]	SQDG (34:1)	16:0/18:1	819.4	
	SQDG (35:0) or SQDG(34:1-OH)	16:0/19:0 or 16:0/18:1-OH	835.5	
	SQDG(28:0)	14:0/14:0 and 12:0/16:0	737.1	
	SQDG(30:0)	14:0/16:0	765.4	
	SQDG(32:0)	16:0/16:0 and 14:0/18:0	793.5	
	SQDG(32:1-OH)	14:0/18:1-OH	807.5	
	SQDG(34:2-OH)	16:0/18:2-OH	833.5	
	SQMG(14:0)	14:0	527.2	
	SQMG(16:0)	16:0	555.3	
	DGTS(34:1)	16:0/18:1		738.5

(C:N) indicates the number of carbon atoms (C) and double bonds (N) in the fatty acyl side chains. Legend: DGTS, diacylglyceryl-N,N,N-trimethylhomoserine; SQDG, sulfoquinovosyldiacylglycerol; SQMG, sulfoquinovosylmonoacylglycerol.
